# Complete Genome Sequence of a Velogenic Newcastle Disease Virus Isolated from an Apparently Healthy Village Chicken in South India

**DOI:** 10.1128/genomeA.00597-14

**Published:** 2014-06-19

**Authors:** Arumugam Uthrakumar, Kumanan Vijayarani, Kathaperumal Kumanan, Srinivasan Bhuvaneswari, Suresh Varma Kuchipudi, Subbiah Elankumaran

**Affiliations:** a Department of Animal Biotechnology, Madras Veterinary College, Tamil Nadu Veterinary and Animal Sciences University, Chennai, India; b School of Veterinary Medicine and Science, University of Nottingham, Sutton Bonington Campus, College Road, Loughborough, Leicestershire, United Kingdom; c Department of Biomedical Sciences and Pathobiology, Virginia-Maryland Regional College of Veterinary Medicine, Virginia Polytechnic Institute and State University, Blacksburg, Virginia, USA

## Abstract

We report the complete genome sequence of a Newcastle disease virus (NDV) isolate, NDV-D1/1998, from an apparently healthy village chicken in South India. This class II, genotype II virus is 15,186 nucleotides in length with unique amino acid variations and was found to be a velogenic pathotype by standard pathogenicity tests.

## GENOME ANNOUNCEMENT

Newcastle disease virus (NDV), or avian paramyxovirus (APMV-1), is a single-stranded, nonsegmented, negative-sense RNA virus belonging to the genus *Avulavirus* (1). NDV infects a wide range of domestic and wild avian species (2). NDV strains have been grouped into class I with a single genotype and class II with at least 18 genotypes (I to XVIII) (3, 4). Greater understanding of the genetic diversity of circulating NDV is required for effective control.

We report here the complete genome sequence of NDV-D1/1998, isolated from an apparently healthy village chicken from the Tamil Nadu State of South India in 1998. The virus was isolated and propagated in specific-pathogen-free (SPF) embryonated chicken eggs. The viral genomic RNA was reverse transcribed and the complete genome sequence was obtained with 32 pairs of primers using Platinum Taq high-fidelity DNA polymerase (Invitrogen). The 5′ and 3′ sequences were obtained by rapid amplification of cDNA ends (5). The PCR products were directly sequenced (Applied Biosystems) in both directions and high-quality contigs were assembled using Lasergene Seqman Pro software (DNASTAR version 11.0). The phylogenetic clustering was made using the maximum likelihood method and the optimum nucleotide model (general time-reversible [GTR]+G+I) as determined by MEGA (6). The new unified proposal for phylogenetic tree construction was adopted (3).

The genome of NDV-D1/1998 was found to be 15,186 nucleotides long. The fusion protein cleavage site sequence of ^112^RRQKRF^117^, the intracerebral pathogenicity index of 1.52, and the mean death time of 56 h confirmed that it is a velogenic pathotype. All animal studies were approved by the ethics committee of the Tamilnadu Veterinary and Animal Sciences University, Chennai. Phylogenetic analysis indicated that NDV/D1-1998 belongs to genotype II with unique amino acid variations. Phosphoprotein (P) protein sequence amino acid variations were noticed at positions E70D, E86D, A92S, D100A, E108Q, F118L, F119L, L120M, F122D, T133K, Y125S, R152G, S163R, N165Q, A173G, Q175D, G176S, T177I, D178Y, V179G, and N180G. These unique notably high ratios of nonsynonymous to synonymous substitutions (*d*_*N*_/*d*_*S*_) might be due to positive selection under vaccine pressure (7), requiring further validation. The 3′ and 5′ untranslated region (UTR) sequences (55 and 114, respectively) and the intergenic sequences were similar to those of other APMV-1 strains. Neutralizing epitopes on the F protein gene are highly conserved. Potential glycosylation sites and hydrophobic regions located at the amino terminus of the F gene are also conserved (8).

Genetic diversity exists between circulating genotype II NDV strains in poultry in South India. NDV/D1-1998 is a highly pathogenic virus isolated from an apparently healthy village chicken. These findings raise questions about the role of village chickens in the epidemiology and evolution of NDV and necessitate further studies. Anecdotal evidence points to greater resistance of indigenous chicken breeds to NDV. Further studies on the genetic and molecular basis of NDV resistance would be helpful to develop selection methods and intervention therapies to improve poultry health globally.

### Nucleotide sequence accession number.

The GenBank accession number of the genome sequence of NDV-D1/1998 is KJ636208.
